# Optimizing Physiotherapeutic Approaches in Parkinson’s Disease Post-spinal Fixation Surgery: A Case Report

**DOI:** 10.7759/cureus.54149

**Published:** 2024-02-13

**Authors:** Prajyot Ankar, Neha P Arya, Tejaswini Fating, Anam R Sasun

**Affiliations:** 1 Community Health Physiotherapy, Ravi Nair Physiotherapy College, Datta Meghe Institute of Higher Education and Research, Wardha, IND

**Keywords:** oswestry disability index, functional independence measure, visual analogue scale, back pain, parkinson’s disease, physiotherapy rehabilitation

## Abstract

Patients with Parkinson's disease (PD) exhibit both a severe neuromuscular disorder and low bone quality at presentation. These issues are made worse by inactivity and a chairbound state. Each and every pathologic and degenerative process that affects the naturally aging spine also affects these individuals. Stooped posture is a symptom of a disease and can easily cause spinal degeneration. PD is associated with many physical abnormalities that cause a unique and specific need for rehabilitation. Patients' experiences highlight the challenges doctors face in diagnosis, treatment, and rehabilitation. This case report details the rehabilitation of a 67-year-old patient with PD who underwent spinal fixation for spinal stenosis and presented with complaints of weakness in both lower limbs. An advanced rehabilitation program was devised, primarily emphasizing strength training to enhance overall functionality. Pre- and post-intervention assessments were conducted, encompassing range of motion (ROM), manual muscle testing (MMT), Oswestry Disability Index, Functional Independence Measure, Lower Limb Functional Scale, and Berg Balance Scale, all of which demonstrated noteworthy improvements in joints ROM, strength, functional independence, balance, and lower limb function. This case report underscores the significance of rehabilitation programs in such cases, highlighting their important role in enhancing overall functioning.

## Introduction

Parkinson's disease (PD), also known as neurodegenerative disease, is a disease that primarily affects the central nervous system (CNS) of the brain. PD, in particular, affects the substantia nigra, a region of the brain located in the midbrain [1]. It is worth noting that PD becomes more common as we get older. Research shows that about 1.5% of people age 60 and older may have PD. Importantly, this means that the risk of PD increases as people get older [2]. PD is a degenerative disease that affects the brain and involves the loss of neurons responsible for dopamine production [3]. It is accompanied by motor symptoms such as tremors, slow movement (bradykinesia), rigidity, and difficulty in controlling the body [4]. The five types of pain experienced by PD patients are musculoskeletal pain, central pain, neuropathic pain, akathisia pain, and dystonia-related pain. Additionally, people with PD often experience symptoms such as thinking and memory problems (cognitive confusion), mood swings, and problems with automatic body functions (autonomic dysfunction). Patients with PD are more likely to experience postoperative complications and unintended revision surgeries following spinal fusion, according to prior research. Furthermore, fusion rates are generally lower and outcomes are worse for PD patients undergoing surgery, particularly for those who have multi-tiered fusion. Although the exact cause is not fully understood, it is believed that a combination of events and environmental influences contributed to its development [5]. Treatment of PD requires a combination of medication and physical therapy to improve the quality of life of people with PD. This report investigates physical therapy after spine surgery in a patient with PD-like symptoms [6]. Management of postoperative complications, especially when neurological disease occurs, causes serious problems in treatment. The combination of spinal pathology and PD-like symptoms in the brain needs to be specialized and treated to return to normal [7].

Of the various pathways that underlie pain related to PD, 40 to 90% of reported symptoms are related to musculoskeletal pain. Complementary therapies, such as physiotherapy and chiropractic adjustments, are recommended for the treatment of musculoskeletal pain. These therapies target joint mobility, motor strengthening, pain management, and postural training exercises. The clinical picture becomes particularly difficult in patients who undergo spine surgery after showing symptoms of PD [8]. It is a serious disease caused by a combination of non-physical symptoms that cause serious complications, especially in spine-related surgical procedures [9]. Interactions between motor control, movement, and post-exercise recovery should be planned and monitored in Parkinson's patients. In this article, we will describe the treatment of a patient with PD characteristics who underwent spine surgery but later developed complications [10]. Postural deformities such as torticollis, anterior neck, Pisa syndrome, and scoliosis are common disabilities in PD and atypical PD [11]. These malformations have a multifactorial pathophysiology that includes muscle stiffness, axial dystonia, myopathy-related weakness, muscle weakness, and spinal cord changes [12]. The impact of these factors varies between patients and specific symptoms. It is a complex disease with many symptoms affecting both motor and non-motor systems. These may include irregular sleep patterns, psychological disorders (such as anxiety and depression), cognitive impairments, and functional disorders (such as constipation or mild gastrointestinal problems) [13].

## Case presentation

A 67-year-old male patient with Grade 2 PD visited the outpatient department, complaining of difficulty walking and maintaining balance. The patient had a history of radiating lower back pain with tingling numbness in bilateral lower limbs, along with weakness in the lower limbs, a year ago. Initially, the symptoms were milder. The patient consulted a local practitioner who prescribed medications, but there was no relief after one month. He visited another hospital with the same complaints, and a magnetic resonance imaging (MRI) was conducted, revealing spinal canal stenosis. Surgery was recommended, and the procedure was performed a week later. Postoperatively, the patient experienced a reduction in pain and tingling numbness, but weakness in the lower limbs persisted. Unfortunately, the patient was not engaged in any rehabilitation. Additionally, two years ago, the patient reported involuntary movements in both upper and lower limbs, coupled with difficulty in maintaining balance. Following the medical consultation, the diagnosis was Grade 2 PD, and ongoing treatment was prescribed. However, no referral was provided for rehabilitation. The patient also has a six-year history of hypertension. Currently, the patient has visited the physiotherapy outpatient department for rehabilitation.

Clinical findings

Consent was obtained prior to the examination. The patient was conscious, cooperative, and oriented to time, place, and person. The patient was examined in a supine position and displayed a mask-like facial expression, and a fully healed scar of approximately 10 cm was noted on the lower back area. During palpation, muscle tone examination indicated a grade 3+ (increased muscle tone) for the upper limbs and a grade 2+ (normal muscle tone) for the lower limbs, as per the tone grading system. Manual muscle testing (MMT) was conducted, revealing reduced muscle strength, particularly pronounced in the lower limbs. All the superficial, deep, and combined cortical sensations were intact. Postural examination showed a forward head posture, shoulder protraction, and a flat back posture. Balance assessment was also conducted using the Berg Balance Scale (BBS), revealing impaired balance. Table 1 shows the examination findings of MMT.

**Table 1:** Manual muscle testing pre-rehabilitation 5+: Complete range of motion (ROM) against gravity with maximal resistance, 4: complete ROM against gravity with moderate resistance, 3+: complete ROM against gravity with minimal resistance, 3: complete ROM against gravity, 3-: some but not complete ROM against gravity, 2+: Initiates motion against gravity, 2: complete ROM with gravity eliminated, 2-: Initiates motion if gravity is eliminated, 1: Evidence of slight contractility but no joint motion, 0: No contraction palpated

Muscle group	Action	Left	Right
Shoulder	Flexors	3-	3-
	Extensors	3-	3-
	Abductors	3-	3-
	Adductors	3	3
Elbow	Flexors	3	3
	Extensors	3	3
Wrist	Flexors	2+	2+
	Extensors	2+	2+
Hip	Abductors	2	2
	Adductors	2	2
	Flexors	2-	2-
	Extensors	2-	2-
Knee	Extensors	3-	3-
	Flexors	2+	2+
Ankle	Plantar Flexors	3-	3-
	Dorsiflexors	2+	2+

Table 2 depicts the range of motion (ROM) findings pre-rehabilitation.

**Table 2:** Range of motion examination pre-rehabilitation

Joints	Movement	Right	Left
Shoulder	Flexion	0-110˚	0-105˚
	Extension	0-35˚	0-35˚
	Adduction	0-115˚	0-120˚
Elbow	Flexion	0-145˚	0-145˚
	extension	145-0˚	145-0˚
Wrist	Flexion	0-65˚	0-60˚
	Extension	0-68˚	0-70˚
Hip	Flexion	0-30˚	0-35˚
	Extension	0-5˚	0-5˚
Knee	Flexion	0-60˚	0-60˚
Ankle	Plantarflexion	0-45˚	0-45˚
	Dorsiflexion	0-10˚	0-10˚

Investigations

**Figure 1:**
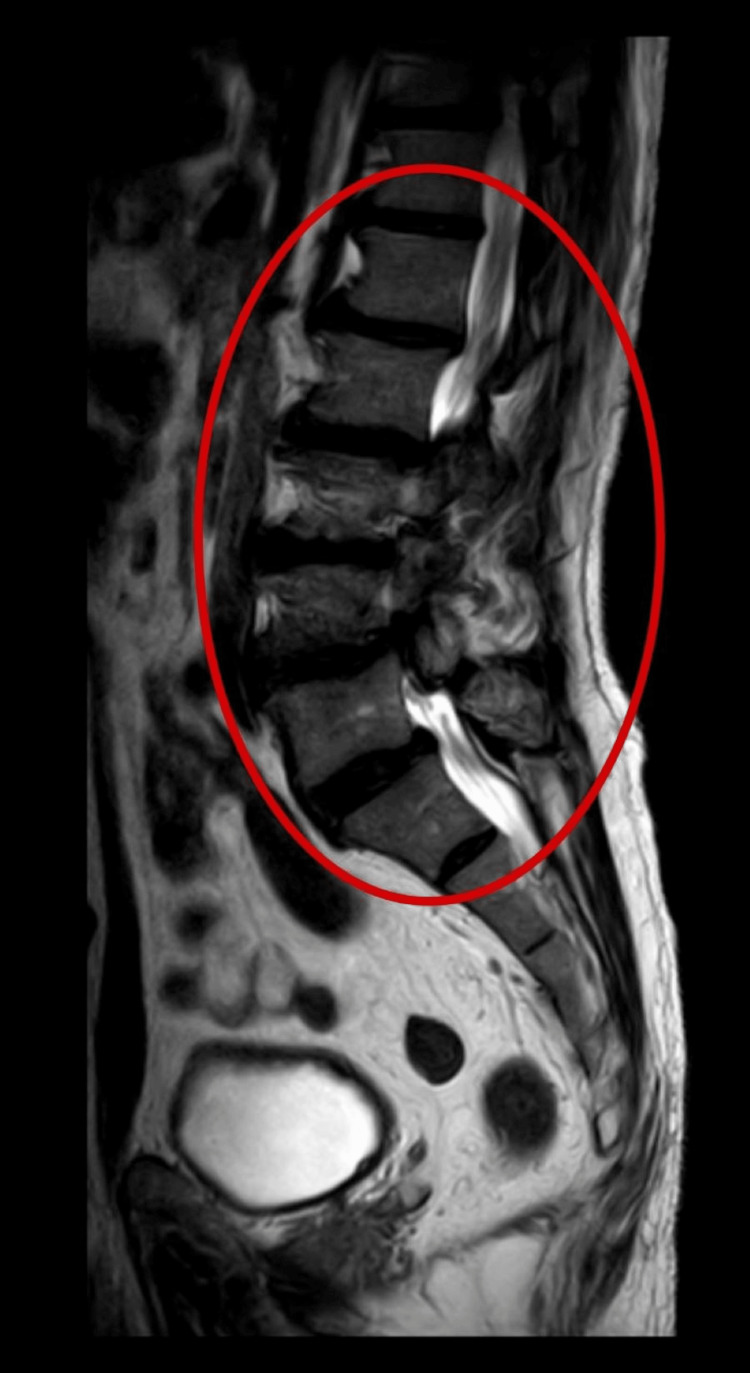
Pre-operative MRI showing compression at L1-L5 (red circle)

The patient underwent an MRI before surgery, which showed the bilateral lateral recess is narrowed and the left-sided disc posterior disc protrusion at the L1-L2 disc level that is indenting over the anterior thecal sac. A widespread disc bulge with e/o causes the spinal canal to shrink at the L2-L3, L3-L4 disc level, obliterating the right and bilateral lateral recesses. At the L4-L5 disc level, there is e/o inferior disc extrusion that results in nearly complete spinal canal obliteration, as shown in Figure 1.

**Figure 2:**
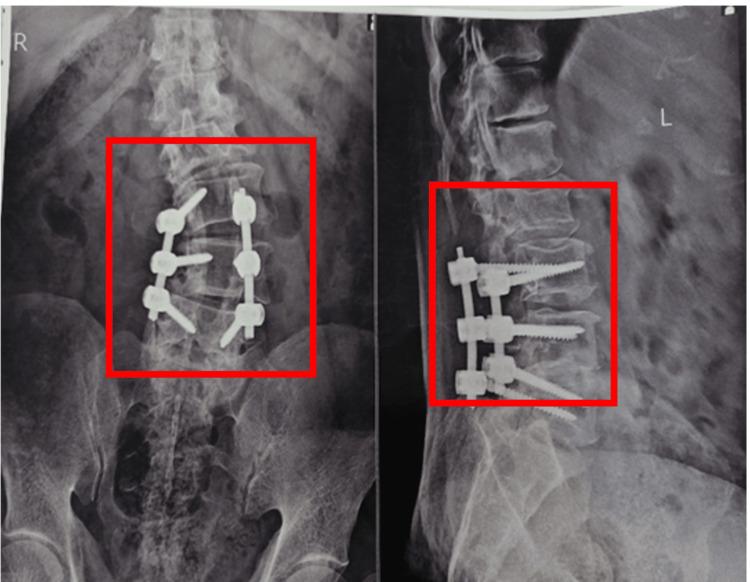
The post-operative X-ray of the lumbar spine (red squares)

Figure 2 depicts a post-operative X-ray showing spinal fixation.

Physiotherapy interventions

A physiotherapy protocol was customized for the patient according to the impairments, and the duration of the rehabilitation program was eight weeks. Table 3 shows the physiotherapy rehabilitation protocol.

**Table 3:** Physiotherapy intervention Reps: repetition, sec: second, Min: minutes, PNF: Proprioceptive Neuromuscular Facilitation, VMO: Vastus Medialis Oblique, LSVT: Lee Silverman Voice Treatment, SLR: Straight Leg Raising

Problem identified	Physiotherapy goals	Interventions
Patient education	Education and counsel to patients	Patient education about his condition and the importance of physiotherapy explanation about the treatment.
Lower extremity weakness	To improve lower extremity muscle strength	Active assisted hamstring curls, dynamic quadriceps, VMO strengthening, progressing to active exercises, active assisted SLR, hip abduction in supine lying progressing to active SLR and hip abduction in side-lying, ankle pumps (10 reps x 2 sets), pelvic bridging (5 reps x 2 sets), wall supported squats (5 reps x 2 sets), PNF rhythmic stabilization to hamstring and quadriceps.
Upper extremity weakness	To improve the strength of the upper extremities.	Shoulder girdle strengthening with ½ kg dumbbell, bicep curls with ½ kg, wrist strengthening with ½ kg, gripping activities (10 reps x 2 sets).
Reduced movement amplitude	To improve movement quality and amplitude	LSVT BIG exercises – floor to ceiling, side to side (10 reps x 2 sets).
Difficulty in walking	To improve gait pattern	Walking with the support of a walker after 2 weeks progressed to unsupported walking, walking on the foot marks, treadmill walking around the obstacles and over the obstacles.
Unable to maintain dynamic balance	To improve dynamic balance	Weight shifting in standing, multidirectional functional reach in standing, heel raises, toe raises, balancing upon the unstable surface (10 reps x 2 sets)
Compromised cardiovascular fitness	To enhance oxygen consumption capacity	Treadmill training for 10 min daily with rest intervals, progressing to 20 mins daily.
To improve resting tremors	To decrease resting tremors	Progressive resistance training programme using weighted vests. (10 reps, 2 sets)

**Figure 3:**
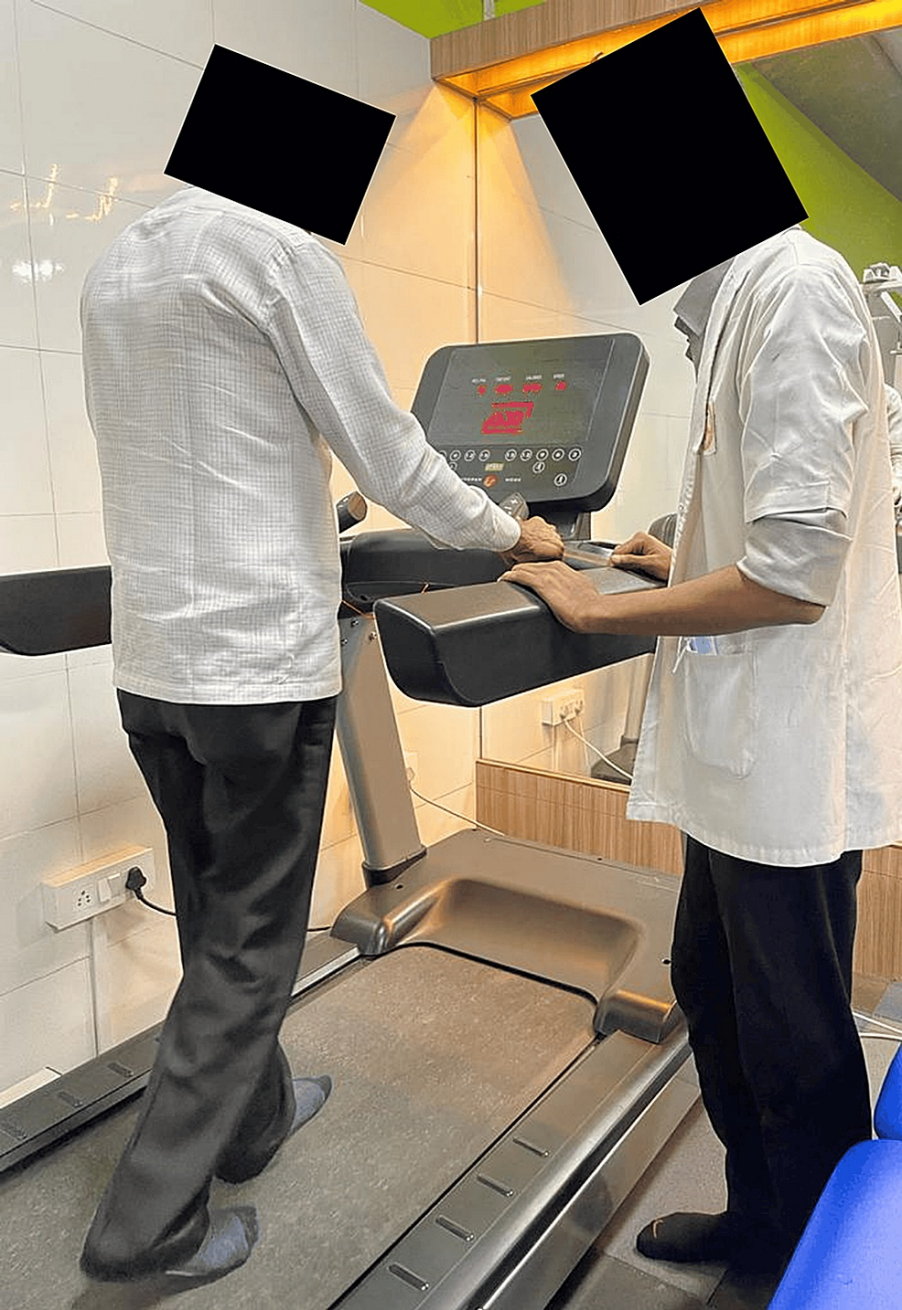
Treadmill training

Figure 3 depicts the patient performing treadmill training.

**Figure 4:**
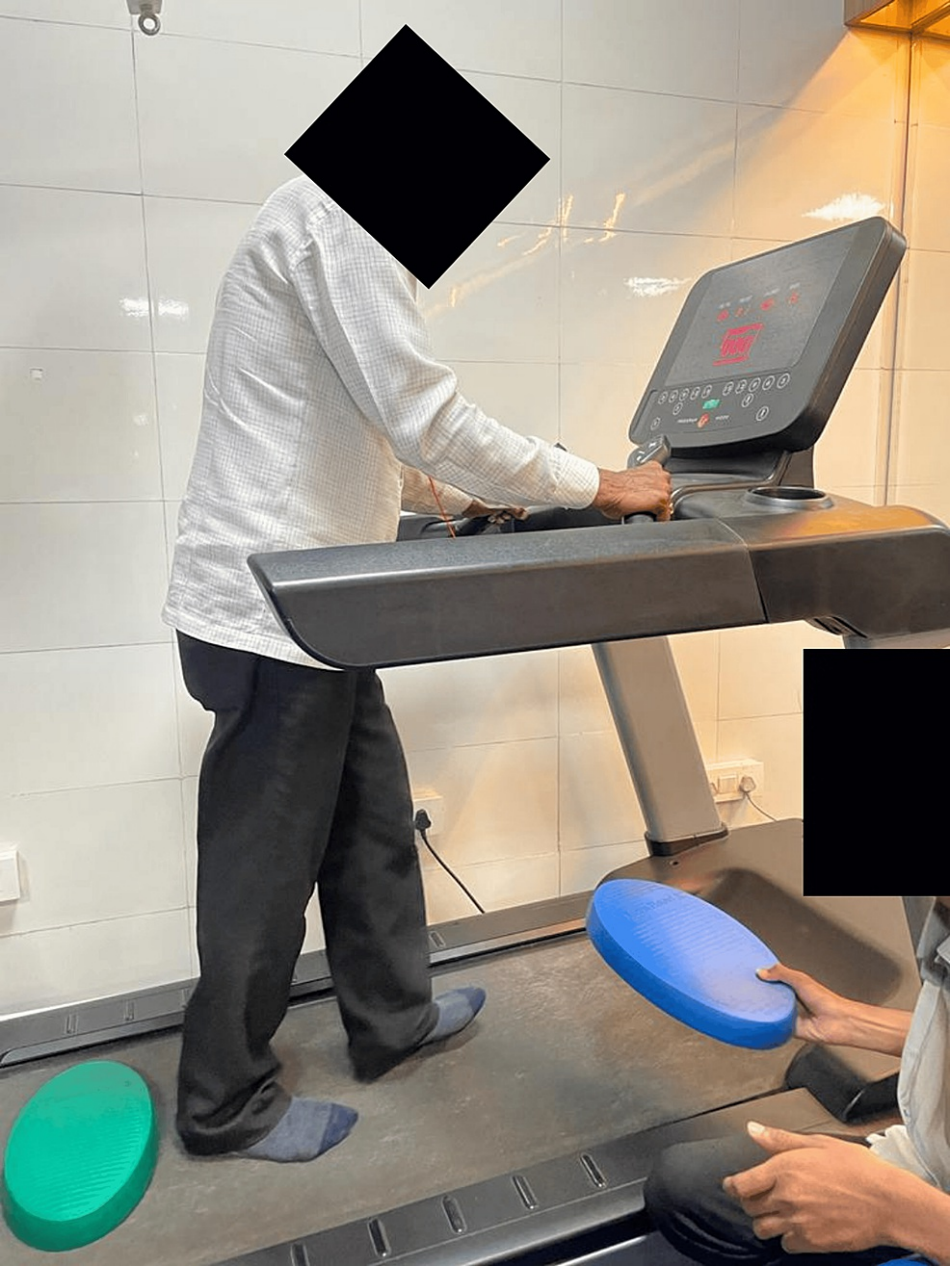
Treadmill training with obstacles

Figure 4 shows the patient walking on the treadmill over the obstacles.

Outcome measures

Assessment for outcome measures was taken pre- and post-rehabilitation on the eighth week, which included examination for ROM, MMT, and the following measures mentioned in Table 4.

**Table 4:** Outcome measures

Outcome measures	Pre-treatment	Post-treatment
Oswestry Disability Index	30	14
Functional Independence Measure	100	119
Lower limb functional scale	32	48
Berg Balance scale	22	40

Table 5 shows MMT findings post-rehabilitation.

**Table 5:** MMT findings post-rehabilitation MMT: Manual muscle testing

Muscle group	Action	Left	Right
Shoulder	Flexors	3	3
	Extensors	3	3
	Abductors	3	3
	Adductors	3	3
Elbow	Flexors	3+	3+
	Extensors	3+	3+
Wrist	Flexors	3	3
	Extensors	3	3
Hip	Abductors	3-	3-
	Adductors	3-	3-
	Flexors	3-	3-
	Extensors	3-	2-
Knee	Extensors	3	3
	Flexors	3	3
Ankle	Plantar Flexors	3	3
	Dorsiflexors	3-	3-

Table 6 shows ROM examination post-rehabilitation.

**Table 6:** ROM examination post-rehabilitation ROM: Range of motion

Joints	Movement	Right	Left
Shoulder	Flexion	0- 170˚	0-172˚
	Extension	0-50˚	0-50˚
	Adduction	0-160˚	0-160˚
Elbow	Flexion	0-145˚	0-145˚
	extension	145-0˚	145-0˚
Wrist	Flexion	0-75˚	0-76˚
	Extension	0-70˚	0-70˚
Hip	Flexion	0-75˚	0-78˚
	Extension	0-15˚	0-20˚
Knee	Flexion	0-135˚	0-135˚
Ankle	Plantarflexion	0-48˚	0-50˚
	Dorsiflexion	0-15˚	0-15˚

## Discussion

The field of PD rehabilitation has been marked by a great deal of diversity. Physical exercise was initially suggested as a therapeutic approach for PD several years ago [14,15]. Since then, rehabilitation therapies have been viewed as a complementary measure to pharmacological and surgical interventions, aiming to optimize functional abilities [16]. Common rehabilitation approaches, including general physiotherapy involving stretching, muscle strengthening, balance, and postural exercises, as well as occupational therapy and treadmill training, are often employed to enhance mobility in individuals with PD [17]. Notably, resistance training is utilized to boost muscle strength, improving gait performance, while stretching helps alleviate the shortening of flexor muscles that contribute to abnormal flexed posture in PD. Lee Silverman Voice Treatment-BIG therapy (LSVT-BIG) emphasizes enhancing movement amplitude to attain larger, swifter, and more precise motions, with the goal of restoring typical movement patterns. By encouraging substantial movements, the therapy aims to counteract hypokinesia by influencing the individual's perception of their movement amplitude [18,19]. Promising results were observed in the restoration of motor functions after just one osteopathic therapy session or a chiropractic manipulation program. Similar to this, musculoskeletal disorders have been treated with a variety of approaches, with a focus on releasing neurological compromise, mobilizing restricted structures, and maximizing the function of afflicted muscles and joints [20].

The physiotherapy management of post-spinal surgery complications in a patient presenting with features of PD is a multifaceted challenge, requiring a tailored and multidisciplinary approach. A customized physiotherapy plan was implemented for eight weeks, which included strength training for both upper and lower limbs, balance training, aerobic training, and LSVT-BIG exercises to improve movement amplitude. A post-rehabilitation evaluation for outcomes was taken at the end of the eighth week, which included the MMT, Oswestry Disability Index, functional independence measure, lower limb functional scale, and BBS, which showed significant improvement likely to be generalized strength, independence for activities of daily living, balance, and lower extremity function. This shows the importance of a planned physiotherapy rehabilitation program in PD. The fact that this was a case report with only one patient observed, the lack of post-treatment X-rays, which made the study observational and the functional outcomes assessed are the study's limitations. Additional research is needed on PD patients who have undergone spinal surgeries.

## Conclusions

This case report underscores the intricate interplay of spinal surgery complications and PD manifestations, emphasizing the necessity for comprehensive rehabilitation. The tailored eight-week physiotherapy intervention, encompassing strength and balance training, aerobic exercises, and LSVT-BIG therapy, yielded significant improvements. The observed enhancements in functional independence, balance, and muscle strength, as reflected in the post-treatment outcome measures, highlight the efficacy of an approach in addressing the complex needs of individuals with coexisting spinal and neurological conditions. This case underscores the importance of integrating rehabilitation strategies into the management of postoperative complications in Parkinson's patients, contributing valuable insights for future clinical practice.
